# Industrial biotechnology of *Pseudomonas putida*: advances and prospects

**DOI:** 10.1007/s00253-020-10811-9

**Published:** 2020-08-13

**Authors:** Anna Weimer, Michael Kohlstedt, Daniel C. Volke, Pablo I. Nikel, Christoph Wittmann

**Affiliations:** 1 Institute of Systems Biotechnology, Saarland University, Campus A1.5, 66123 Saarbrücken, Germany; 2 The Novo Nordisk Foundation Center for Biosustainability, Technical University of Denmark, Lyngby, Denmark

**Keywords:** *Pseudomonas putida*, Microbial cell factory, KT2440, EDEMP cycle, Bacterial chassis, Biocatalysis, Biotransformation, Metabolic engineering, Synthetic biology, Bioeconomy, Lignin, PHA

## Abstract

**Abstract:**

*Pseudomonas putida* is a Gram-negative, rod-shaped bacterium that can be encountered in diverse ecological habitats. This ubiquity is traced to its remarkably versatile metabolism, adapted to withstand physicochemical stress, and the capacity to thrive in harsh environments. Owing to these characteristics, there is a growing interest in this microbe for industrial use, and the corresponding research has made rapid progress in recent years. Hereby, strong drivers are the exploitation of cheap renewable feedstocks and waste streams to produce value-added chemicals and the steady progress in genetic strain engineering and systems biology understanding of this bacterium. Here, we summarize the recent advances and prospects in genetic engineering, systems and synthetic biology, and applications of *P. putida* as a cell factory.

**Key points:**

*• Pseudomonas putida advances to a global industrial cell factory.*

*• Novel tools enable system-wide understanding and streamlined genomic engineering.*

*• Applications of P. putida range from bioeconomy chemicals to biosynthetic drugs.*

**Electronic supplementary material:**

The online version of this article (10.1007/s00253-020-10811-9) contains supplementary material, which is available to authorized users.

## Introduction

**Fig. 1.**
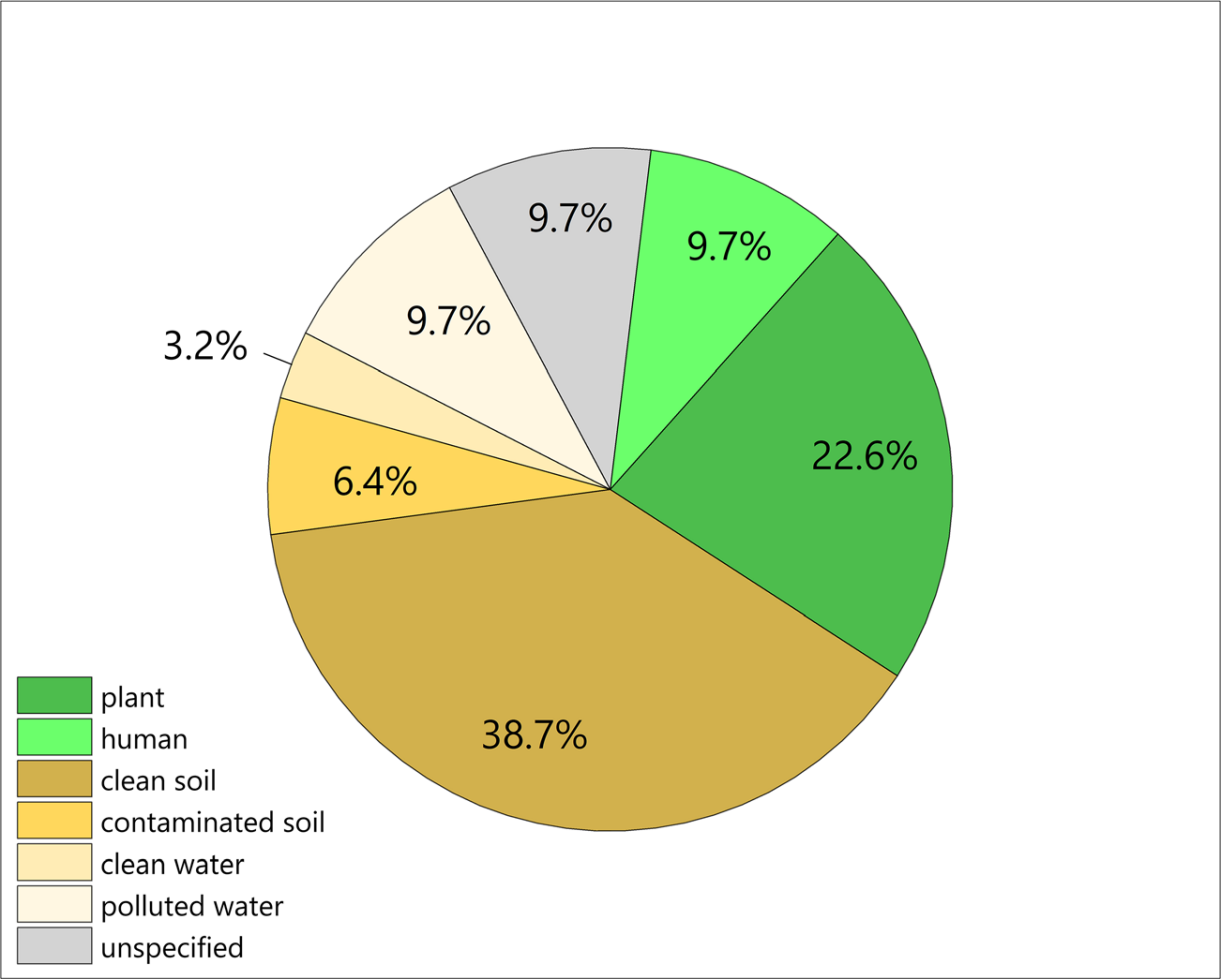
Isolation, source, and distribution of *P. putida* strains with full genome sequence available. The *P. putida* strains have been isolated from soil, polluted soil, water, polluted water, and/or wastewater and other unspecified sources (Data from *Pseudomonas* Genome DB and NCBI BioSamples Database, accessed: 05/20/2020; see Tables S2 for further information.)

**Fig. 2.**
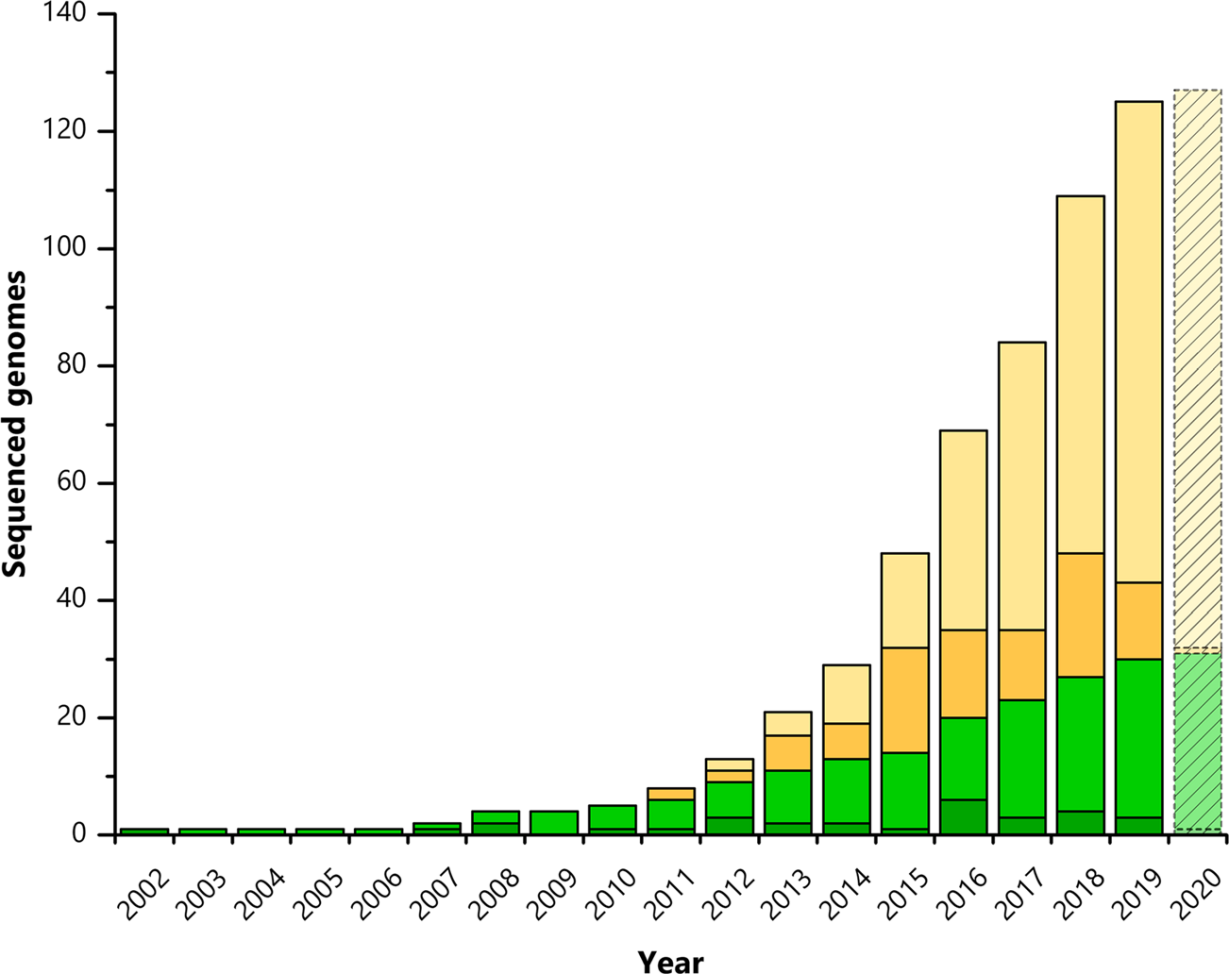
Typical carbon flux distribution throughout central carbon metabolism of glucose-grown *Pseudomonas putida*. The *P. putida* features a predominant ED pathway, coupled with an incomplete EMP pathway and activities of the PP pathway. The C3 intermediates, pyruvate (PYR), and glyceraldehyde-3-P (G3P), are recycled back via the gluconeogenic operation of the EMP pathway, a network topology called EDEMP cycle (Nikel et al. 2015). Respective enzyme-coding genes (orange), redox and energy cofactors (light purple)

*Pseudomonas putida* is a Gram-negative, rod-shaped bacterium, frequently isolated from waters, plants, and soils (in particular, polluted sites) (Fig. 1). The heterogeneity of P. putida’s natural environment is reflected by its opportunistic and undemanding nutritional capabilities, rapid growth, as well as robustness upon challenges with oxidative stress and toxic chemicals. Initiated by the discovery of the potential of *P. putida* in biodegradation of xenobiotics in the 1960s (Nakazawa 2002), the acquisition of knowledge about the genetics, biochemistry, and physiology of this microbe has been continuously progressing over the last five decades. This led, inter alia, to the decryption of the complete genomic repertoire (Belda et al. 2016; Nelson et al. 2002) (Fig. 2) and the construction of genome-scale metabolic models for in silico simulations and data mapping (Nogales et al. 2020; Puchałka et al. 2008). Moreover, an ever growing number of tools for systems-level profiling, targeted genetic and genome manipulations are being developed (Cook et al. 2018; Martínez-García and de Lorenzo 2017). This increasing knowledge and technology, together with the intrinsic biochemical capabilities of the bacterium, offers vast industrial application potential. Among others, representative members of the species have been identified as plant growth-promoting microbes (Glick 2012), bioremediation agents (Dvořák et al. 2017), and hosts for industrial bio-manufacturing, including the production of bulk and specialty chemicals (Nikel and de Lorenzo 2018; Poblete-Castro et al. 2012a; Tiso et al. 2014), natural products, such as rhamnolipids, terpenoids, polyketides and non-ribosomal peptides (Loeschcke and Thies 2015), and biopolymers (Mozejko-Ciesielska et al. 2019; Rehm and Valla 1997; Salvachúa et al. 2020a). The most prominent and well-studied TOL plasmid-free strain *P. putida* KT2440 has been especially considered as a microbial host for biotechnological application, due to its biosafety status (National Archives and Records Administration 1982). Other strains for industrial use include the solvent-tolerant strains *P. putida* S12, DOT-T1E, and VLB120 (Kohler et al. 2013; Udaondo et al. 2012; Wierckx et al. 2005), the plant growth-promoting strains BIRD-1 and UW4 (Duan et al. 2013; Matilla and Krell 2018), the phenol-degrading H strain (Müller et al. 1996), strain LS46 for production of medium-chain-length polyhydroxyalkanoates (mcl-PHA) (Sharma et al. 2014), as well as *P. putida* F1, isolated from soil and developed into a bioremediation agent (Choi et al. 2003). Over the past years, research on *P. putida* has immensely progressed. Recent prominent innovations include high-resolution metabolic flux analysis (Kohlstedt and Wittmann 2019; Nikel et al. 2015; Sasnow et al. 2016), synthetic remodeling of the metabolic core machinery (Sánchez-Pascuala et al. 2019), production of green chemicals from lignin feedstocks (Kohlstedt et al. 2018; Salvachúa et al. 2020a), and the synthesis of new-to-nature products (Martinelli and Nikel 2019). In this review, we provide an overview on the most recent and impacting developments in the field, which are shifting the industrialization of P. putida onto a new level.

**Fig. 3.**
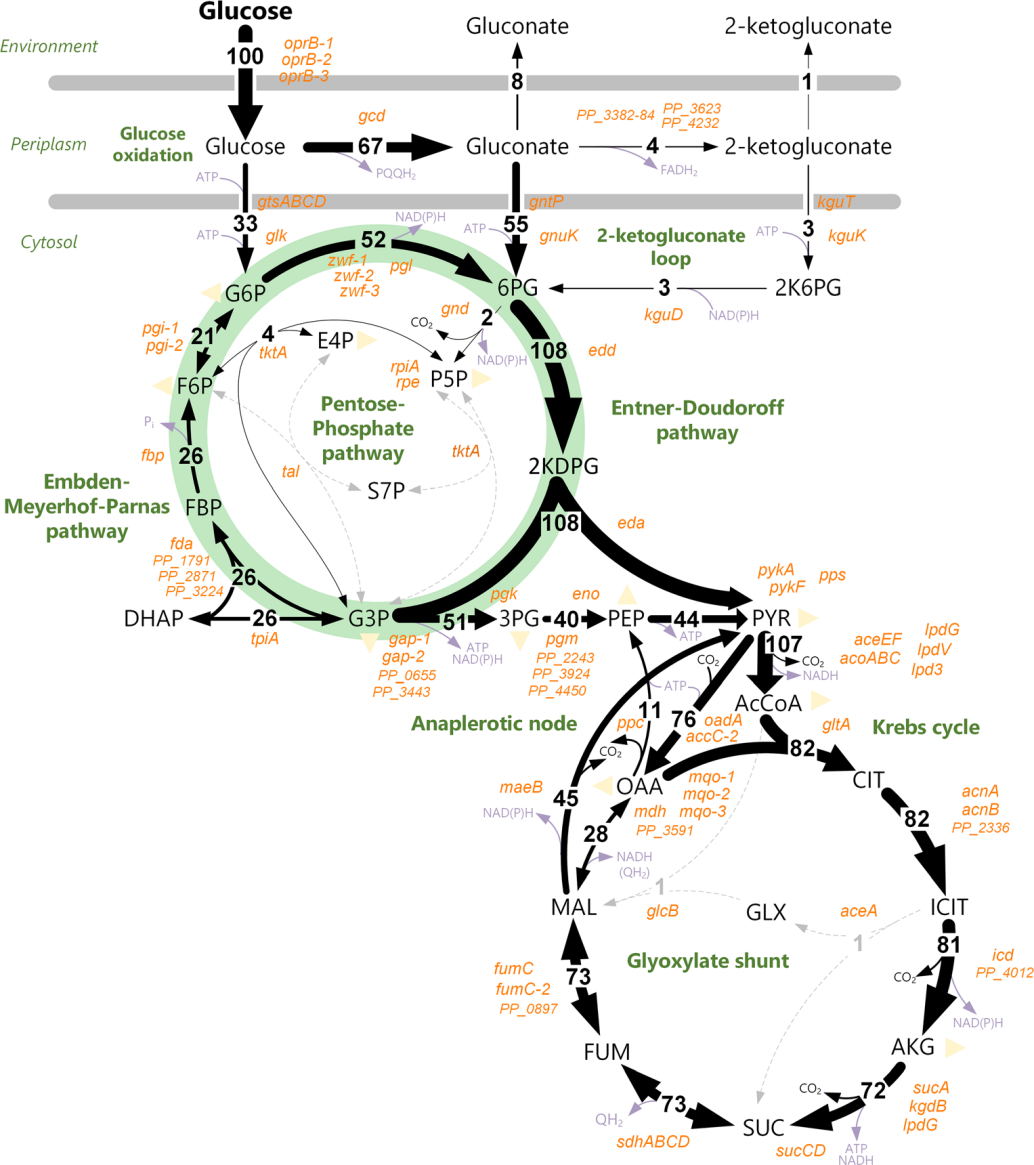
Available genome sequences of *P. putida* strains*.* Accumulated number of publicly available full (light green) and draft genome sequences (light yellow) and newly available full (dark green) and draft genome sequences (dark yellow), published in the indicated year (Data from *Pseudomonas* Genome DB and NCBI accessed: 05/20/2020; see Tables S1 and S2 for further information)

## Regulation of core carbon and energy metabolism

The *P. putida* is often found in contaminated environments, which speaks in favor of a remarkable adaptation capability of the microbe to adverse conditions (Silby et al. 2011). Its atypical cyclic core metabolism, controlled on a redox demand, plays a key role to enable the high endurance observed (Chavarria et al. 2013; Nikel et al. 2015) (Fig. 3).

After entry into the periplasmic space, glucose is either internalized into the cytoplasm or oxidized in the periplasm. Gluconate (GLN) and subsequently 2-ketogluconate (2KG) are formed via the latter oxidation pathway. Both acids can be transported into the cytoplasm followed by phosphorylation into 6-phosphogluconate (6PG) and 6-phospho-2-ketogluconate (2K6PG). Hence, there are three different entry pathways of glucose into the core metabolism, converging at the level of 6PG (del Castillo et al. 2007). The oxidation pathways enable *P. putida* to circumvent the direct ATP-costly glucose uptake via an ABC transporter (GtsABCD) and to partially uncouple ATP formation from NADH formation. Two electrons are released in each oxidation step from periplasmic glucose to GLN and 2KG, which is coupled to ATP generation via the ATP synthase (Ebert et al. 2011). Recently, it has been shown that glucose-grown cells generate an ATP surplus, whereas the oxidation pathway contributes significantly to the supplied ATP (Kohlstedt and Wittmann 2019). In addition, *P. putida* possesses an incomplete Emden-Meyerhof-Parnas (EMP) pathway, due to the absence of the key glycolytic enzyme 6-phosphofructo-1-kinase (Pfk). The central intermediate 6PG is further catabolized almost exclusively through the Entner-Doudoroff (ED) pathway, resulting in the two C3 intermediates pyruvate (PYR) and glyceraldehyde-3-P (G3P). A dominant fraction of the former enters lower catabolism. However, a significant part (approximately 10–20% under balanced growth conditions) is recycled back to hexoses via the gluconeogenic EMP pathway, an amphibolic architecture termed as EDEMP cycle (Nikel et al. 2015). The NADPH yield is coupled to the reaction catalyzed by glucose-6-P 1-dehydrogenase (G6PDH) and, hence, depends significantly on the extent of recycling and the fraction of glucose phosphorylated via glucokinase (GLK). The ability to adjust the NADPH formation at the expense of ATP is a key factor to oxidative stress endurance in *P. putida*. This feature provides uttermost benefit in redox-demanding biocatalytic processes, which has also been shown to be important for the evolvability of novel catabolic pathways in this bacterium (Akkaya et al. 2018). Notably, *de novo* refactoring of the central carbon metabolism of KT2440 could be demonstrated by implementation of a functional linear glycolysis, based on the EMP pathway (Sanchez-Pascuala et al. 2017; Sánchez-Pascuala et al. 2019), which endows cells with novel tailor-made properties.

## Recent advances in systems biology of *Pseudomonas*

A major challenge in metabolic engineering and synthetic biology is the understanding of the underlying metabolic and regulatory networks (Nogales et al. 2020), including the complex links between newly introduced and native biochemical pathways (Pandit et al. 2017). An important prerequisite to breed better cell factories is to understand the interactions occurring between the many cellular components at different functional and hierarchical levels, i.e., genome, transcriptome, proteome, metabolome, and fluxome. Recent systems biology studies, using and combining various omics technologies and collecting systems-level data in online databases, have greatly advanced our view on *P. putida* and have proven valuable to design more robust and efficient cell factories in a sophisticated manner (Poblete-Castro et al. 2013; Thompson et al. 2019).

### Genomics and metabolic reconstructions

First published in 2002 and revisited in 2016, the full genome sequence of *P. putida* KT2440 has shed light on the diverse transport and metabolic systems (Belda et al. 2016; Nelson et al. 2002). Moreover, the pangenome of *P. putida* has been studied and revealed 3386 conserved genes belonging to the core genome and comprising genes related to ED and pentose phosphate (PP) pathway, proline and arginine metabolism, aromatic compound degradation, as well as a vast collection of nutrient transporters (Udaondo et al. 2016). Notably, besides sharing 85% of the coding regions with *Pseudomonas aeruginosa*, undesirable biotechnological traits, i.e., key virulence factors, exotoxins and type III secretion systems, are lacking in *P. putida* (Udaondo et al. 2016). Since 2010, the number of published genome sequences of *P. putida* has significantly increased (Fig. 2, Tables S1 and S2)*.* Up to now, 28 complete genomes and 88 draft *P. putida* genomes can be accessed via the *Pseudomonas* genome DB (pseudomonas.com), allowing for the identification of new features for industrial application.

Reconstructed from sequence information, several genome-scale metabolic models (GSMM) are available for *P. putida* (Belda et al. 2016; Henry et al. 2010; Nogales et al. 2008; Oberhardt et al. 2011; Puchałka et al. 2008; Sohn et al. 2010; Yuan et al. 2017). Very recently, the GSMM iJN1462 of *P. putida* KT2440 was published (Nogales et al. 2020), which is comparable to high-quality *Escherichia coli* models in size and level of detail. With the increasing number of *P. putida* genomes available, these models can facilitate the identification of strains and enzymes most suitable for the respective industrial application and drive the design of superior producers (Buschke et al. 2013; Choi et al. 2019; Poblete-Castro et al. 2013).

### Transcriptomics and proteomics

As suggested by the elevated number of open-reading frames (450 in total), encoding transcriptional factors and 24 alternative sigma subunits of the RNA polymerase, gene expression in *P. putida* is tightly regulated at the transcriptional level (Martínez-Bueno et al. 2002). Transcriptomic profiles of *P. putida* KT2440 grown on different substrates have been studied in response to environmental perturbations (glucose, fructose, succinate, citrate, ferulic acid, serine, and glycerol) (D'Arrigo et al. 2019; Kim et al. 2013; Nikel et al. 2014), as well as profiles during the shift between alternative carbon sources (Sudarsan et al. 2014). Furthermore, recent transcriptomic studies under osmotic, oxidative, and imipenem stress (Bojanovič et al. 2017) and increased heavy metal concentrations (Peng et al. 2018) added knowledge about the stress response mechanisms in *P. putida*. Notably, during exposure to solvents, a general stress response initiated the expression of molecular chaperons and other stress resistance proteins during exposure to solvents, which is favorable for biocatalysis in a hydrophobic milieu (Dominguez-Cuevas et al. 2006). More latterly, the transcriptional response during nitrogen-limited *mcl*-PHAs synthesis on gluconate (*de novo* fatty acid synthesis pathway) and oleic acid (*β*-oxidation pathway) as the sole carbon sources was studied*.* Several genes were suggested as possible targets for strain improvement towards more efficient production (Mozejko-Ciesielska et al. 2018)*.* Moreover, by characterizing laboratory evolved *P. putida* KT2440, utilizing genome re-sequencing and qRT-PCR, the metabolic and transcriptional regulatory bases of ethylene glycol metabolism in *P. putida* were uncovered (Li et al. 2019). Another interesting recent study mimicked repeated glucose shortage under large-scale heterogeneously mixed fermentation to investigate the metabolic and transcriptional response of *P. putida* KT2440 to starvation (Ankenbauer et al. 2020). A transcriptional regulation program linked to the intracellular pool of 3-hydroxyalkanoates, precursors of PHAs, was identified to be probably associated with accession of cellular PHA, amino acids, and glycogen to quickly restore ATP levels and the adenylate energy charge, a desired feature for large-scale bioreactors. In addition, 20 repeatedly upregulated ATP-consuming genes were promoted as possible technical targets. These genes are likely to be associated with long-term cellular programs and are superfluous in a bioreactor environment where glucose starvation is temporary (Ankenbauer et al. 2020).

Proteomics have been used to analyze *mcl*-PHA biosynthesis (Fu et al. 2015; Możejko-Ciesielska and Mostek 2019; Nikodinovic-Runic et al. 2009; Poblete-Castro et al. 2012b). Moreover, studies on post-transcriptional regulatory events, with vanillin and butanol as the sole carbon source, reported an increased abundance of enzymes of the tricarboxylic acid (TCA) cycle, indicating that this cycle is the main pathway for energy production when glucose is unavailable to *P. putida* (Simon et al. 2014; Simon et al. 2015)*.* In addition, an increased abundance of efflux pumps and a rearrangement of transporter patterns have been shown during toluene exposure (Wijte et al. 2011). Recently, shotgun proteomics identified the oxoprolinase-encoding *oplBA* genes, responsible for the undesired hydrolysis of valerolactam *P. putida*, leading to a more efficient production process (Thompson et al. 2019). Besides, application of proteomics analysis and genome re-sequencing enhanced the understanding of the plastic monomer 1,4-butanediol metabolism in *P. putida* KT2440 (Li et al. 2020).

### Metabolomics and fluxomics

The cyclic operation of the EDEMP pathway in *P. putida* has been proven by the combination of isotope labeling experiment, followed by quenching, extraction, and LC-MS analysis of intracellular intermediates of the central metabolism, and *in vitro* enzyme assays (Nikel et al. 2015; Sasnow et al. 2016). Lately, a high-resolution flux map of glucose-grown *P. putida* KT2440 was obtained from parallel labeling experiments using [1-^*13*^C]-, [6-^*13*^C]- and 50% [^*13*^C_6_]-glucose, and straightforward GC–MS analysis of hydrolyzed cell pellets (Kohlstedt and Wittmann 2019). By then, GC–MS-based flux studies in *P. putida* were extremely limited, due the unresolvable cyclic network using common flux approaches. This was circumvented by deriving the labeling patterns for G6P and F6P from an extended approach that additionally included labeling information from glycogen, lipopolysaccharides, and peptidoglycan. The novel strategy displays a breakthrough for systems biology studies of *P. putida* (Mendonca et al. 2020) and related strains (Dolan et al. 2020). At a large, metabolic flux analysis has been foremost conducted with glucose as the sole carbon source (Kohlstedt and Wittmann 2019; Nikel et al. 2015; Sasnow et al. 2016); thus, the cyclic operation of the EDEMP pathway was also demonstrated using glycerol (Beckers et al. 2016; Dolan et al. 2020) as the sole carbon source. Furthermore, these metabolic flux studies revealed an inactive glyoxylate shunt during growth on the glycolytic substrate glucose (Sasnow et al. 2016), whereas the glyoxylate shunt was found active during growth on the gluconeogenic substrate glycerol (Beckers et al. 2016) or acetate (Dolan et al. 2020). A recent study has taken up this topic by using ^*13*^C-metabolomics to elucidate a carbon portioning of glucose and benzoate within the metabolic network. Carbon atoms derived from glucose were cycled through the EDEMP and PP pathway, whereas benzoate was preferentially catabolized through the TCA cycle and glyoxylate shunt and the atoms derived from this substrate did not enter the EDEMP nor the PP pathway. This segregation was shown to sustain biosynthetic flux demands (Kukurugya et al. 2019) and appears an interesting feature for some bio-production processes, where a multi-substrate feeding strategy can be advantageous.

### Multi-omics integration

Several studies have combined different omics approaches towards an even more complete picture. Integrating transcriptomics, proteomics, and metabolomics, the differences between nutrient conditions on PHA and biomass production in *P. putida* KT2442 were studied. A significantly different cellular rewiring was observed for conditions under single nutrient limitation compared with nutrient co-limitation (Poblete-Castro et al. 2012b). Moreover, carbon catabolite repression (CCR) was investigated, using a combination of metabolic, transcriptomic, and constraint-based metabolic flux analyses. It was demonstrated that central metabolic fluxes of cells grown in succinate and glucose as carbon sources are regulated by CCR and this regulation contributes to the organization and optimization of the metabolism and growth (La Rosa et al. 2015). Interestingly, in follow-up studies, CCR and the resulting metabolic rearrangements were also shown to be advantageous for growth in complete LB medium (La Rosa et al. 2016; Molina et al. 2019). Another example study investigated the cellular response of *P. putida* KT2440 on the transcriptomic, proteomic, and metabolic levels in a chemostat cultivation. The C4 alcohol *n*-butanol, an attractive biofuel, was used either as sole carbon source or together with glucose. Based on ^*13*^C-fluxome analysis, carbon portioning was observed: glucose was directed into the ED and PP pathways, and *n*-butanol fueled the TCA cycle, when both substrates were consumed. In addition, an unknown *n*-butanol degradation pathway was discovered with the help of transcriptomic and proteomic analyses (Vallon et al. 2015).

## Recent advances in genetic engineering

The meanwhile large toolbox for genetic and genome engineering eases the construction workflow of reliable *P. putida* production strains. In recent years, the set of modular vectors of the Standard European Vector Architecture (SEVA) platform has turned out to be a priceless resource for construction of recombinant *P. putida* strains (Martínez-García et al. 2015; Martínez-García et al. 2019; Silva-Rocha et al. 2012). One traditionally established molecular biology resource for analyses and manipulations of *P. putida* genomes are fully synthetic derivatives of Tn*5*- and Tn7-based transposon vectors (Choi et al. 2005; Martínez-García et al. 2014a). The insertion of the transposon into the genome can be either random (e.g., mini-Tn5 vectors) or site specific (e.g., mini-Tn7 vectors). Due to the randomness of insertion, Tn*5*-based transposon vectors have been applied for the generation of random mutant libraries (Martinez-Garcia and de Lorenzo 2012), as well as for random insertions of entire gene clusters (Martínez-García et al. 2014a) with subsequent screening for superior phenotypes. Tn7-based transposon vectors were used to create a library of promoters and translational couplers (Zobel et al. 2015) and to optimize production of secondary metabolites (Loeschcke et al. 2013). Some of the mini transposon vectors are furthermore compatible with the SEVA format (Martínez-García et al. 2014a; Zobel et al. 2015). Inherently, these systems require a selection marker. Flp recombinase target-flanked antibiotic resistance determinants have been used for precise excision of selection markers with the corresponding recombinase Flp. Thus, one copy of the Flp recognition target (FRT) site will always remain after excision, limiting the repeated use of these procedures (Nikel and de Lorenzo 2013b). Subsequently, efficient genome editing methods that do not leave selection markers nor foreign DNA sequences, such as CRISPR/Cas9 technologies (Aparicio et al. 2018; Kim et al. 2020; Pham et al. 2020; Wirth et al. 2020; Zhou et al. 2020), DNA recombineering (Aparicio et al. 2020a; Aparicio et al. 2020b; Choi et al. 2018; Choi and Lee 2020), and homologous recombination-based DNA editing (Galvão and de Lorenzo 2005; Graf and Altenbuchner 2011; Martínez-García and de Lorenzo 2011), have been developed and applied. The most widespread homologous recombination-based technique for genome engineering in *P. putida* involves two rounds of recombination. First, the suicide plasmid pEMG or derivatives, bearing recognition sequences for the intron encoded I-*Sce*I homing endonuclease from yeast, is integrated into the genome. In the second, counter-selection step double-stranded breaks (DSB) are introduced in the genome by conditional expression of the I-*Sce*I homing endonuclease encoded by a helper plasmid*.* DSBs are repaired by homologous recombination across the regions of sequence flanking the ends of the break, allowing for gene deletion, insertion, and replacement (Martínez-García and de Lorenzo 2011). Thus far, curing of the helper plasmid requires repetitive passaging of clones in antibiotic-free medium and is therefore a rather time-intensive step. Just recently, synthetic control of helper plasmid replication enabled self-curing of the plasmid in a mere overnight cultivation (Volke et al. 2020a). Moreover, fluorescent markers encoded by both the suicide plasmid and the helper plasmid eased the screening process and amenability of this approach was demonstrated by 23 kb genomic deletions, resulting in the streamlined strain SEM10 (Volke et al. 2020a). More recent innovative developments deal with recombineering methods, such as RecET-based markerless recombineering system for deletion and integration of large-sized genes and clusters (Choi and Lee 2020), efficient single-stranded recombineering by using a thermoinducible system (Aparicio et al. 2020a), as well as CRISPR/Cas9 technologies. The latter was utilized for efficient curing of helper plasmids (Wirth et al. 2020), counterselection of infrequent mutations created through recombineering (Aparicio et al. 2018), metabolic engineering for PHA bioconversion from ferulic acid (Zhou et al. 2020), as well as CRISPR interference-mediated gene regulation (Batianis et al. 2020; Kim et al. 2020). The incredible pace of development of new tools leaves no doubt that there will be precise CRISPR-based technologies soon, speeding up genomic manipulations even further. Additionally, several tools initially developed in *E*. *coli* bear high potential to be transferred to *P. putida* (Martínez-García and Lorenzo 2019).

Furthermore, industrial processes require defined gene expressions by natural or synthetic promoters. Besides the already mentioned constitutive promoter libraries for chromosomal expression (Elmore et al. 2017; Kohlstedt et al. 2018; Zobel et al. 2015), another interesting study demonstrated the achievability of a wide range of chromosomal expression in *P. putida* (Elmore et al. 2017). Moreover, a variety of inducible promoters, both synthetic and natural, have been characterized in *P. putida*, as covered elsewhere (Martínez-García and de Lorenzo 2017). Recently, cell density-dependent auto-inducible promoters based on the RoxS/RoxR Quorum Sensing system of *P. putida* have been developed and tested in the KT2440 strain. Theses promoter systems without the need of induction are especially interesting for industrial processes where protein expression independent of the addition of an inducer is desired to reduce the metabolic burden during exponential growth phase (Meyers et al. 2019). Furthermore, *rrn* operons have demonstrated a strong preference for biosynthetic gene cluster integration in *P. putida*, due to stable integration and strong expression by the native promoters (Domröse et al. 2019). Finally, it was recently demonstrated that fine-tuning of metabolic pathways via targeted proteolysis enables a new control layer of engineered pathways (Calles et al. 2019; Volke et al. 2020b) .

## *Pseudomonas putida* as a bacterial host for industry: default beneficial built-in properties

A microbial host needs to meet several performance criteria and quality requirements for industry, such as easy handling, predictable and reproducible production behavior, and natural robustness to ease process establishment. In fact, *P. putida* already features many desirable properties by default. Foremost, there is vast basic knowledge about *P. putida*, due to a tremendous research volume—a prerequisite for a simplified workflow for any further metabolic engineering. In a nutshell, the lessons learned about *P. putida* are the following: (a) The bacterium exhibits fast growth, high biomass yields, low to no by-product secretion and low maintenance demands (Poblete-Castro et al. 2012a). (b) The *P. putida* naturally sustains a surplus production of ATP and high rates of NADPH regeneration, due to EDEMP overflow metabolism on hexoses (Kohlstedt and Wittmann 2019; Nikel et al. 2015) and the metabolic routes can be also rewired to fuel the EDEMP cycle in a bottom-up fashion to enable NADPH overproduction from other gluconeogenic substrates, such as glycerol (Beckers et al. 2016). (c) The vast regulatory apparatus empowers *P. putida* with a high flexibility to quickly adjust to steady changing conditions (Kukurugya et al. 2019; Sudarsan et al. 2014), which is especially desirable in a large-scale bioreactor with heterologous microenvironments (Ankenbauer et al. 2020). (d) The microbe possesses a versatile catabolism of carbon sources. On top of that, the substrate spectrum of *P. putida* was successfully expanded towards the utilization of sucrose (Löwe et al. 2017), L-arabinose (Meijnen et al. 2008), D-cellobiose (Dvořák and de Lorenzo 2018), D-xylose (Bator et al. 2020; Dvořák and de Lorenzo 2018; Le Meur et al. 2012; Meijnen et al. 2008; Meijnen et al. 2009), phenol (Vardon et al. 2015), and ethylene glycol (Franden et al. 2018). Therefore, cheap, renewable feedstocks with a high level of impurities, such as glycerol, a by-product from biodiesel production (Poblete-Castro et al. 2020b), and aromatic compounds derived from lignin (Kohlstedt et al. 2018; Vardon et al. 2015), can be exploited for production of value-added chemicals. The secretion of outer membrane vesicles (OMVs) has been described as an additional mechanism for extracellular nutrient acquisition (Eberlein et al. 2019; Salvachúa et al. 2020b). (e) A high tolerance against physicochemical stress, chemical stresses (e.g., heavy metal zinc (Peng et al. 2018), cadmium (Manara et al. 2012), arsenic (Cánovas et al. 2003)), solvents (Kusumawardhani et al. 2018), and oxidative stress has been retraced inter alia to an efficient regulation machinery (Kim and Park 2014), secretion systems, *trans*-isomerization of the cell membrane, and changes in head group composition of cell membrane phospholipids (Ramos et al. 2015). This opens up the possibility to use biphasic systems (Verhoef et al. 2009; Wierckx et al. 2005), as well as directed laboratory evolution experiments where toxic compounds are present (Li et al. 2019). (f) Moreover, *P. putida* is naturally endowed with an elevated GC content (61–63%) (Udaondo et al. 2016), allowing to use it for heterologous expression of genes from GC-rich microbes bearing secondary metabolite biosynthetic gene clusters, such as actino- and myxobacteria (Chai et al. 2012; Gross et al. 2006a; Gross et al. 2006b; Kimura et al. 1996; Mi et al. 2014). All these properties render *P. putida* an excellent host for industrial biotechnology.

## *Pseudomonas putida* as a bacterial host for industry: creation of tailor-made synthetic properties

**Fig. 4.**
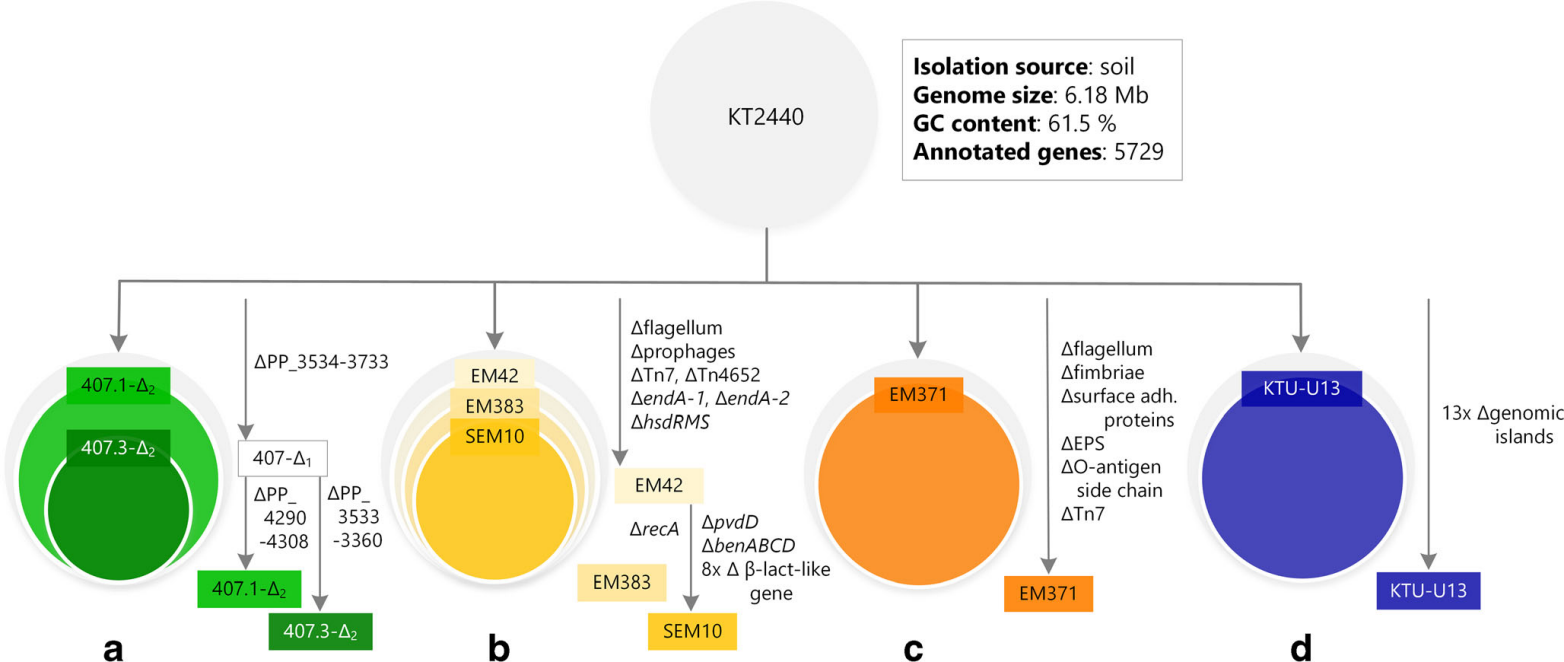
Genome-reduced strains of *P. putida* KT2440 constructed to date. The diagram represents the genealogy of parent strain KT2440 and its genome reduced derivatives. (**a**) Cyclic, random large-scale deletions using a mini-Tn*5* transposon combined with the FLP-*FRT* recombination system. A first deletion round resulted in strain 407-Δ_1_ (gray box; Δ*PP_3534*-*PP_3733*), and subsequent deletion rounds resulted in strains 407.1-Δ_2_ (light green; Δ*PP_4290*-*PP_4308*) and 407.3-Δ_2_ (dark green; Δ*PP_3533*-*PP_3360*). (**b**) Large target deletions, using the homologous recombination-based I-*Sce*I methodology of the flagellar operon (Δ*PP_4329*-*PP_4397*), 4 prophage elements, Tn*7* transposase and Tn*4652* transposon, deoxyribonucleases I encoded in *endA-1* and *endA-2*, and the *hsdRMS* operon (Δ*PP_4740*-*PP_4742*) encoding a I DNA restriction-modification system resulted in strain EM42 (yellow). Further deletion of *recA* resulted in strain EM383, and elimination of eight *β*-lactamase(-like) genes, *pvdD* (involved in siderophore formation), and the *benABCD* gene cluster led to strain SEM10. (**c**) Large target deletions, utilizing the I-*Sce*I methodology, of genes encoding flagellum, fimbriae, surface adhesion proteins, exopolysaccharides, *O*-antigen side chain, and envelope-associated compounds and Tn*7*-like transposase operon (orange). (**d**) Large target deletion of 13 genomic islands using an *upp*-based counter-selection system resulted in strain KTU-U13 (blue). Reduced genome sizes, depicted as smaller cycles than that of strain KT2440, are not drawn to scale of genome reduction

For the reasons mentioned previously, *P. putida* has made a reputation for being a promising microbial chassis for the bioindustry. Several research groups have started to expand its natural capacity even further. Streamlined *P. putida* strains that harbor reduced genomes offer improved characteristics, such as increased ATP and NAD(P)H availability, superior growth properties, and elevated resistance to oxidative stress (Table 1; Fig. 4).

**Table 1.** Top-down approaches for constructing streamlined *P. putida* strains

Strain	Genome reduction	Deletion	Characteristics	Reference
407.1-Δ_2_	~ 4.4%	Δ*PP_3534*-*PP_3733*; Δ*PP_4290*-*PP_4308*	Similar or better growth than the wild-type strain	Leprince et al. (2012)
407.3-Δ_2_	~ 7.4%	Δ*PP_3534*-*PP_3733*; Δ*PP_3533*-*PP_3360*	Similar or better growth than the wild-type strain	Leprince et al. (2012)
EM42	~ 4.3%	Δflagellar operon, Δ*endA-1*, Δ*endA-2*, Δprophages, ΔTn*7*, ΔTn*4652*, Δ*hsdRMS*	Superior growth properties; increased energy charge, higher NADPH level; improved genomic stability and plasmid structural stability than strain KT2440	Martínez-García et al. (2014b)
EM383	~ 4.3%	Δ*recA*	Derivative of EM42; improved genomic stability	Martínez-García et al. (2014b)
SEM10	~ 4.8%	Δβ-lact(-like) genes Δ*pvdD* Δ*benABCD*	Derivative of EM42; Increased susceptibility towards β-lactam antibiotics, enhanced biosafety, reduced autofluorescence, facilitated use of 3-methylbenzoate as an inducer (no colored by-products)	Volke et al. (2020a)
EM371	~ 4.7%	Δflagellum Δfimbriae Δsurface adherence proteins ΔEPS ΔO-antigen side chain ΔTn*7* Δprophage 4	Improved accessibility of the cell surface, improved genomic stability, improved resistance to UV than strain KT2440	Martinez-Garcia et al. (2020)
KTU-U13	~ 4.1%	Δgenomic islands	Similar growth, increased plasmid stability, potential improvement of expression level of heterologous proteins	Liang et al. (2020)

Naturally, *P. putida* is a strict aerobic bacterium, due to the absence of fermentative pathways and the inability to use alternative electron acceptors. By tackling this problem, the performance of *P. putida* in large bioreactors can be enhanced. Synthetic fermentation pathways and nitrate/nitrite respiration were introduced in strain KT2440, which resulted in higher survival under anoxic conditions (Nikel and de Lorenzo 2013a; Steen et al. 2013). In addition, anoxic cultivation of *P. putida* was demonstrated in the anodic compartment of a bioelectrochemical system (BES), using redox mediators and an anode as extracellular electron sink to balance the intracellular redox and energy factors (Lai et al. 2016; Schmitz et al. 2015; Yu et al. 2018). The novel field of electro-biotechnology of *P. putida* provides an excellent starting point for high-yield production of sugar acids without the need for oxygen (Lai et al. 2019). Furthermore, an interesting development has managed to engineer the morphology of *P. putida* to render the lifestyle from a planktonic towards a biofilm-based one, which can exhibit a higher tolerance to harsh reaction conditions during biotransformations (Benedetti et al. 2016). This is an unique property of *Pseudomonas* species, where the whole-cell catalyst can be manipulated to adopt a spatial configuration that greatly facilitates the purification of extracellular products (Volke and Nikel 2018). Moreover, autodisplay of enzymes can be advantageous in some whole-cell biocatalytic approaches: Being connected to the cell as a matrix, the surface-displayed biocatalyst is readily stabilized and purified and substrates and products do not necessarily need to cross the membrane barrier (Jose 2006). Recently, an improved autotransporter-based surface display of an esterase and a *β*-glucosidase was demonstrated using *P. putida* KT2440, utilizing the native *P. putida* OprF signal peptide (Tozakidis et al. 2020). Very promising, in order to make the cell surface more accessible to the outer medium, 230 genes were deleted from the parent strain *P. putida* KT2440 (∼ 4,7% genome reduction size), including surface adhesion proteins, exopolysaccharides, fimbriae, the *O*-antigen side chain, the flagellum, and other envelope-associated components (Martinez-Garcia et al. 2020). The resulting strain EM371 displays a platform strain for artificial adhesins, which was already used for the successful display of designer protein scaffolds on the surface of *P. putida* cells, opening up the possibility to engineer artificial cellulosomes (Dvořák et al. 2020a). Under harsh bioprocess conditions, however, the degree of surface exposure may need to be further tweaked, since the larger cell surface contact area of EM371 also leads to an increased sensitivity to external stressors (Martinez-Garcia et al. 2020).

## Bioproduction and industrial application

**Fig. 5.**
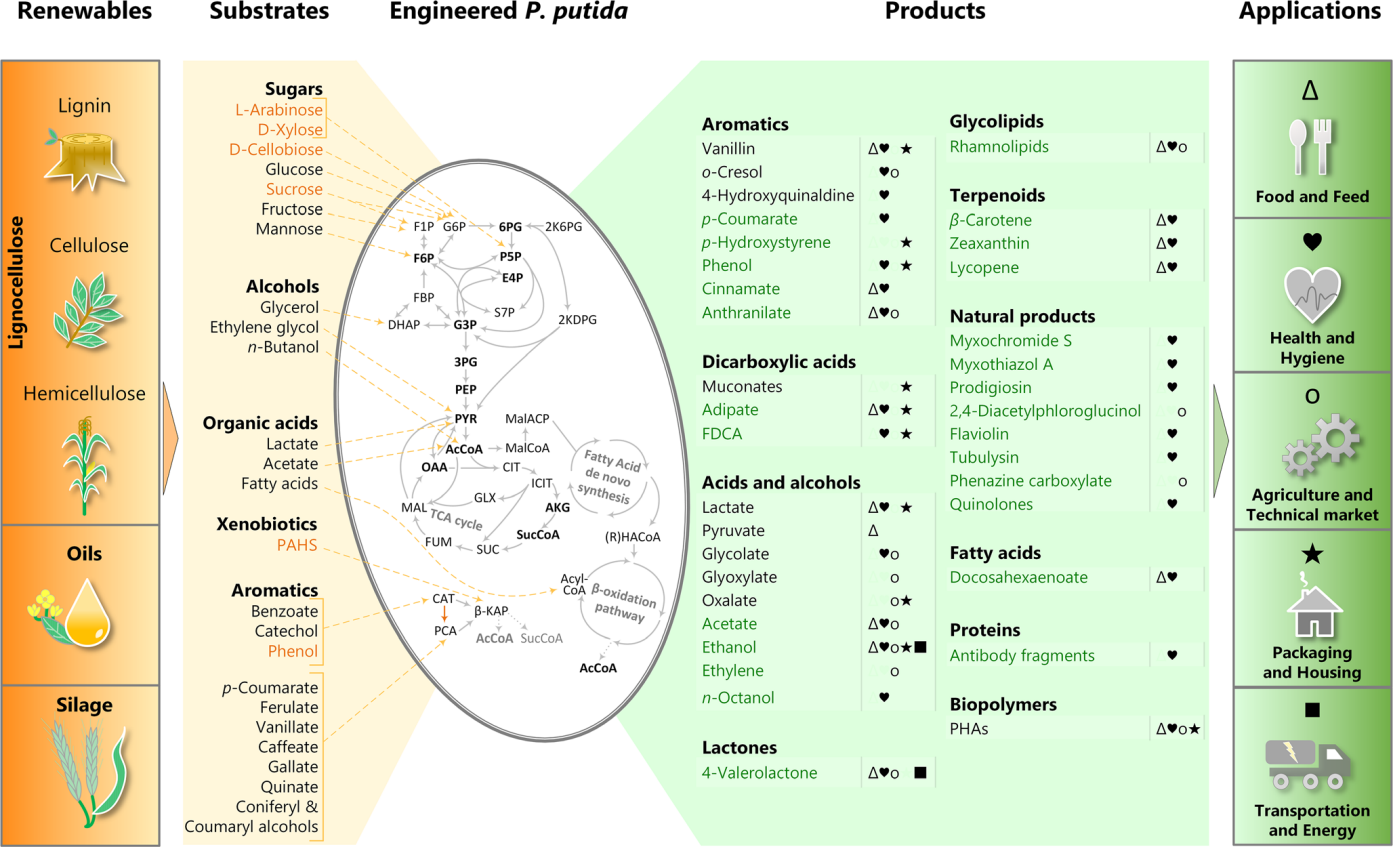
Bioproduction using engineered *P. putida* strains. Substrates generated from renewable feedstocks, such as lignocellulose, oils and silage can be used to produce value added products for application in the food and feed (Δ), health and hygiene (♥), packaging and housing (★), transportation and energy sector (■), and for agriculture and technical application (o). Entry points of the respective substrates in the metabolism (dashed orange line), new to *P. putida* substrates (orange) and products (green)

Not surprisingly, works on biotechnological application of this bacteria evaluated and conceived *P. putida* for its use in bioremediation (de Lorenzo 2008; Jiménez et al. 2002; Shim and Yang 1999; Udiković-Kolić et al. 2012), as a biocontrol agent and plant growth-promoting bacteria (Gouda et al. 2018; Matilla and Krell 2018). Furthermore, the application spectrum of *P. putida* has grown considerably over the last few years and *P. putida* has proven to be an excellent bacterial host to produce polymers, bulk chemicals, drugs, and high-price specialties (Fig. 5).

### Polyhydroxyalkanoates

Endopolymeric *mcl*-PHAs are naturally synthesized by *P. putida* as a carbon and energy storage compound under specific conditions, such as carbon excess during nutrient limitation (N, O_2_, P, S) (Poblete-Castro et al. 2012b). The family of PHA polyesters is one of the best-known product classes studied in *Pseudomonas* species (Prieto et al. 2016). PHAs offer an excellent alternative for petroleum-based plastics, due to their biodegradability and competitive material properties, such as biocompatibility, lack of toxicity, insolubility, and thermostability (Mozejko-Ciesielska et al. 2019). The structural composition of PHAs can be adjusted by feeding of precursors (Wang et al. 2010), cultivation conditions, and strain engineering (Tripathi et al. 2013). The companies Kaneka (Japan), Telles (USA), Jiangsu Nantian (China), Tianjin GreenBio (China), Tepha (USA), DSM (The Netherlands), Biomer Biotechnology (Germany), Bio-on (Italy), Polyferm (Canada), and Biomatera (Canada) report to produce PHA polymers on an industrial scale using *P. putida* (Poltronieri and Kumar 2017). The global PHA market size is expected to grow from US$ 57 million in 2019 to US$ 98 by 2024, wherein the major constrain for growth is the cost competitiveness to conventional polymers (MarketsandMarkets™ 2019). The *P. putida* can produce *mcl*-PHAs from a broad spectrum of carbon sources, giving the opportunity to use cheap renewable feedstocks, such as crude glycerol from biodiesel production (Poblete-Castro et al. 2014), plant-derived fatty acids (Cerrone et al. 2014), food waste (Follonier et al. 2014), lignin-derived aromatics (Liu et al. 2017; Salvachúa et al. 2020a), and even non-degradable plastic waste, PET monomers (Kenny et al. 2008). In recent studies, the first production of PHAs from cellobiosan, levoglucosan (Linger et al. 2016), and cellobiose (Dvořák et al. 2020b) was reported. The *P. putida* EM42-expressing *bglC* from *Thermobifida fusca*, encoding *β*-glucosidase, accumulates *mcl*-PHA and concomitantly secretes xylonate, a platform chemical (Dvořák et al. 2020b). Such co-production of valuable bioproducts from low-cost substrates along with PHA represents a good opportunity to mitigate the overall PHA production costs (Li et al. 2017). Besides feedstock costs, downstream processing and product recovery is an important cost factor for the industrial production of intracellular compounds, which needs to be addressed to make the industrial application economical feasible. Some of the conventional methods include solvent extraction and chemical digestion. However, these methods are also marked with possible environmental drawbacks, high costs, or degradation of the polymer. Recently, a recovery of nearly 94% of the synthesized *mcl*-PHA after 3 h could be shown with cell disruption through a programmable cell lysis system in *P. putida* KT2440 engineered to respond to osmotic state (Poblete-Castro et al. 2020a). Taken together, recent achievements will pave the way to further reduce process costs at the level of raw material selection and downstream processing.

### Alginates

Even though less studied, *P. putida* can produce exopolymeric alginates (Conti et al. 1994) and dehydration has been suggested as the general signal for the production of this polysaccharides (Chang et al. 2007). Alginate is a common additive to cosmetics and foods and has many medical applications. However, up to now, alginate production has been mainly studied in the pathogenic *P. aeruginosa* and there has been no commercial application associated to these efforts (Liu et al. 2019; Valentine et al. 2020).

### *Cis,cis*-muconic acid

The arsenal of oxidoreductases, mono- and dioxygenases encoded in the genome of *P. putida*, enables this bacterium to degrade a variety of aromatic compounds found in the renewable feedstock lignin (Nogales et al. 2017). The aromatics are channeled through catabolic funneling via the *β*-ketoadipate (*β*-KA) pathway converging in only a few central intermediates (i.e., catechol, protocatechuate (PCA)), which are ring-cleaved and further converted into TCA cycle intermediates. One intermediate of this pathway, *cis,cis*-muconic acid (MA), is a very promising molecule, as it can be used as a starting material for the synthesis of value-added chemicals, such as caprolactam, terephthalic and adipic acid, as well as a bulk chemical in polymer reactions for the production of muconic homo- and copolymers (Khalil et al. 2020). Upon disruption of the degradation route at the level of muconate cycloisomerase (CatB), a stoichiometric conversion of aromatic substrates into MA can be achieved (van Duuren et al. 2011). Several studies reported successful MA production using *P. putida* KT2440 (Johnson et al. 2017; Johnson et al. 2016; Kohlstedt et al. 2018; Salvachúa et al. 2018; Sonoki et al. 2018; van Duuren et al. 2012; van Duuren et al. 2011; Vardon et al. 2015). In a landmark study, the biotransformation of a broad range of aromatic monomers, namely protocatechuate, ferulate, coniferyl alcohol, vanillin, caffeate, *p*-coumarate, 4-hydroxybenzoate (feeding into the PCA branch) and catechol, benzoate, and phenol (fueling the catechol branch), into MA was demonstrated. For this purpose, the PCA branch of *β*-KA pathway was connected to the catechol branch by heterologous expression of PCA decarboxylase encoded by the *aroY* gene from *Enterobacter cloacae*, with a simultaneous disruption of the natural PCA degradation route catalyzed by protocatechuate 3,4-dioxygenase (PcaHG). As a result and important proof of concept, 0.7 g L^−1^ MA was produced from alkaline pretreated liquor—a waste stream from bioethanol production (Vardon et al. 2015). However, for some aromatic monomers tested, accumulation of intermediates was observed, indicating an inefficient conversion to MA (Vardon et al. 2015). Subsequently, improvement of MA production efficiency from aromatics of the PCA branch was achieved by increasing PCA decarboxylase activity via co-expression of accessory proteins *ecdBD* from *E. cloacae* (Johnson et al. 2016) and by tackling this bottleneck on the regulatory level by eliminating CCR (Johnson et al. 2017). Since all lignin-derived aromatics eventually merge at the level of the highly toxic intermediate catechol, this node is of interest as metabolic engineering target. In a seminal study, during processing of toxic catechol, cellular energy limitation was identified as a major challenge (Kohlstedt et al. 2018). To overcome this drawback on the operation side, implemented specific feed pauses during fed-batch fermentation allowed the regeneration of cellular energy levels and a final MA titer of 64.2 g L^−1^ from catechol was obtained, exceeding previously reported values by more than tenfold. A second strategy reported in this study created superior KT2440 cell factories. Several rounds of metabolic engineering resulted in an improved platform strain for catechol conversion: increased catechol 1,2-dioxygenase activity by implementing a copy of the *catA2* gene (natively directly downstream of the *catA* gene under control of the native P_cat_ promoter). Thereby, a strongly improved tolerance to catechol and a higher catechol degradation rate could be achieved. The developed process was transferred to pilot-scale–producing MA at the kilogram scale with 98% purity. Subsequently, by heterologous expression of a phenolhydroxylase, the strain is now able to additionally convert phenol into MA and cresols into methylated derivatives of MA. The final strain produced 13 g L^−1^ MA from a softwood lignin hydrolysate (Kohlstedt et al. 2018). Engineered strains for MA are no longer able to grow on aromatics; therefore, glucose is commonly used as the growth substrate. Recently, a study reported glucose-independent MA production using engineered KT2440. This strain was able to produce small amounts (20 mg L^−1^) of MA from depolymerized softwood lignin (Sonoki et al. 2018). Moreover, MA production was also achieved using glucose as the sole substrate (Bentley et al. 2020; Johnson et al. 2019).

### Adipic acid and nylon 66

**Fig. 6.**
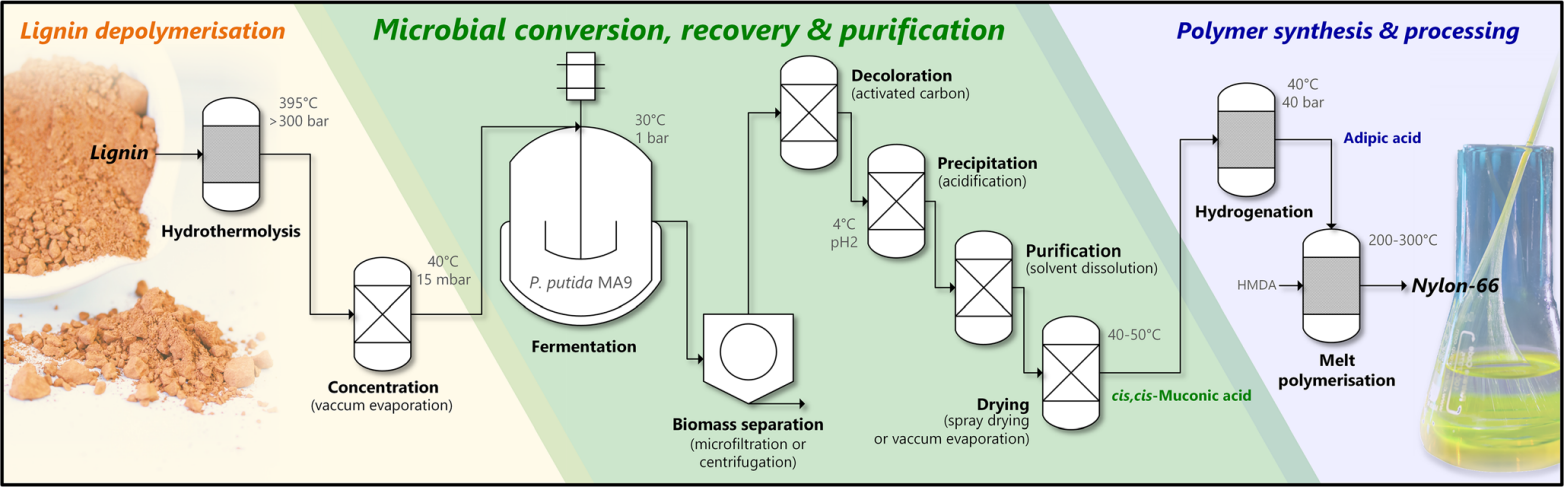
Cascaded biochemical and chemical route for nylon-66 production using metabolically engineered *P. putida*. Lignin depolymerization, microbial conversion of aromatic monomers to MA, subsequent product recovery and purification, hydrogenation to adipic acid and final polycondensation with hexamethylenediamine (HMDA) to nylon-66 (Kohlstedt et al. 2018)

To showcase the entire value chain from lignin to bio-based nylon, MA underwent hydrogenation to adipic acid, a precursor for commercial nylon-66 (Kohlstedt et al. 2018; Vardon et al. 2015; Vardon et al. 2016). The combined chemical and biochemical process is displayed in Fig. 6. A recent limited life cycle assessment concluded the feasibility of the bio-based production of adipic acid from softwood lignin-derived aromatics based on *P. putida* as the whole-cell biocatalyst (van Duuren et al. 2020) and even possible offset credits were promoted for the bioethanol biorefinery, if the lignin in the their wastewater is no longer burnt but converted to the value-added product adipic acid instead (Corona et al. 2018). Purification of MA produced by *P. putida* from lignin model compounds by activated carbon treatment was demonstrated with a good recovery at > 97% purity. Subsequently, Pd/C-aided catalytic hydrogenation of MA yielded bio-based adipic acid (Vardon et al. 2015).

Recently, the feasibility of direct *de novo* synthesis of adipic acid from lignin model compounds was demonstrated using *P. putida* KT2440 (Niu et al. 2020). To this end, the pathway design profited from the C6 structure of the *β*-KA pathway intermediate 3-ketoadipoyl-CoA, i.e., there is no need to exploit a condensation reaction between an acetyl-CoA and a succinyl-CoA unit as previously reported for adipic acid biosynthesis using glucose- or glycerol-grown *E. coli* (Cheong et al. 2016; Yu et al. 2014; Zhao et al. 2018).

### 2,5-Furandicarboxylic acid

The important platform chemical hydroxymethyl furfuraldehyde (HMF) can be derived from mono- and polysaccharides and pre-treated biomass (Dutta et al. 2012). Further catalytic oxidation of HMF yields the building block 2,5-furandicarboxylic acid (FDCA), which has potential applications in the production of plasticizers, polyamides, and polyesters (Corbion 2020; Sousa et al. 2015). One polymer of particular interest is polyethylene furanoate (PEF), a copolymer of ethylene glycol and FDCA, which represents an ideal substituent for polyethylene terephthalate (PET) in packaging, due to its exceptional thermal and superior barrier properties (Sousa et al. 2015). It may also serve as precursor for poly(1,4-cyclohexanedimethylene furandicarboxylate (PCF) (Wang et al. 2018). The global FDCA market is growing with an assessment at US$ 243.9 million in 2020 and is expected increase to US$ 321.3 million by the end of 2026 (QY Research 2020). Companies, such as Stora Enso (Stora Enso 2019), Novamont (Novamont 2019), and Avantium (2023: 5000 tons/year) (Avantium 2020) are currently entering the market with the construction of (pilot) plants for the production of biomass-derived FDCA. Whereas, chemocatalysis is the predominant route for the production of FDCA, which exhibits some disadvantages, such as costly metal catalysts, utilization of organic solvents, and the requirement of high temperature and pressure (Yuan et al. 2020). However, biocatalytic approaches are hindered by the toxic biological effects of furan aldehydes, attributable to ROS-induced oxidative stress (Allen et al. 2010; Almeida et al. 2009). Engineered *P. putida* KT2440 was able to grow on HMF and furfural as the sole carbon source after heterologous expression of the catabolic HMF and furfural gene clusters from *Burkholderia phytofirmans* (Guarnieri et al. 2017). For biotransformation of HMF to FDCA, *P. putida* S12 was chosen as a host, due to its natural tolerance to chemical stressors and the availability of endogenous aldehyde dehydrogenases to oxidize HMF to its corresponding, less toxic carboxylic acid (Koopman et al. 2010). By episomally expressing the oxidoreductase encoding *hmfH* gene from *Cupriavidus basilensis* HMF14, the efficient production of FDCA was reported for the first time, using a whole-cell transformation (Koopman et al. 2010). Subsequently, FDCA production was optimized by episomal coexpression of *hmfH*, aldehyde hydrogenase *adh* and *hmfT1* transporter gene from *C. basilensis* HMF14 in *P. putida*, resulting in high, reproducible production rates (Wierckx et al. 2012). Recently, chromosomal co-integration of *hmfH* and *hmfT1* in *P. putida* S12 was demonstrated by coupling *λ*-Red-mediated recombineering with CRISPR*/*Cas9 technology (Pham et al. 2020). Very promising, application of the patent dealing with FDCA production from HMF, using *P. putida* is filed by a subsidiary of Corbion, a company which is active in the industrial production of PEF derived from FDCA (Corbion ; Wierckx et al. 2012).

### Aromatics

The natural tolerance and ability to metabolize aromatics have been exploited for the region-selective bioproduction of 3-methylcatechol (Hüsken et al. 2001), the bioproduction of *o*-cresol from toluene in a two-phase 1-octanol/water system (Faizal et al. 2006), as well as epoxidation of styrene in a water/octanol two-liquid phase system (Blank et al. 2008). Other produced building blocks include pyruvate and lactate from *p*-coumarate and benzoate (Johnson and Beckham 2015). Moreover, the bioconversion of ferulic acid to the flavor agent vanillin was achieved with resting cells (Graf and Altenbuchner 2014). Interestingly, in this study, the sole deletion of the vanillin dehydrogenase gene (*vdh*) did not suppress degradation of the later and one hitherto unknown molybdate-dependent oxidoreductase was found to probably complement the *vdh* inactivation. The natural tolerance of *P. putida* towards aromatic compounds has not only been exploited for bioconversions and -transformations, but also for *de novo* synthesis of aromatic compounds originating from shikimate pathway intermediates. Using the solvent-tolerant *P. putida* S12 strain production of cinnamic acid (Nijkamp et al. 2005), phenol in a biphasic fed batch cultivation system with octanol (Wierckx et al. 2005) and *p*-hydroxystyrene in a water/1-decanol phase (Verhoef et al. 2009) were reported. Anthranilate, *p*-coumaric acid, and *para*-hydroxybenzoic acid were synthesized from glucose by KT2440-derived strains (Calero et al. 2016; Kuepper et al. 2015; Yu et al. 2016).

### Biosurfactants

Rhamnolipids are biodegradable and low toxic biosurfactants. The activity of biosurfactants enhances the solubility of hydrophobic molecules in water by decreasing the surface tension (Rehm 2009). Potential applications can be found in food industry, cosmetics, cleaning agents, biocontrol, and soil remediation (Fracchia et al. 2014; Loeschcke and Thies 2015). The *P. putida* is able to produce short-chain rhamnolipids after heterologous integration of the *rhlAB(C)* operon from *P. aeruginosa* (Tiso et al. 2017; Wittgens et al. 2011) and long-chain rhamnolipids after expression of *rhl* genes from *Burkholderia glumae* (Wittgens et al. 2018)*.* Wherein, the chain length depends on the respective expressed *rhl* genes rather than the available 3-hydroxy fatty acids, a property which can be used for the production of tailor-made rhamnolipids (Wittgens et al. 2018). The chemical company Evonik Industries (Essen, Germany) announced the first industrial production of rhamnolipids in 2016 (Evonik Industries 2016), followed by an issued patent in 2017 for rhamnolipid production from *n*-butane by *P. putida* KT2440 (Thum et al. 2017).

### Terpenoids

The high tolerance of *P. putida* against the toxicity of intermediates or terminal products has proven to be advantageous to produce terpenoids, one of the largest and structurally diverse groups of natural compounds. Taking the natural resistance into advantage, the successful de *novo* production of geranic acid, a monoterpenoid with several reported antibiotic activities and uses as flavor and fragrance agent, could be shown. This study also revealed a remarkably higher resistance of *P. putida* to more than 6-fold higher product concentrations in comparison to *E. coli* and *Saccharomyces cerevisiae* (Mi et al. 2014). Furthermore, oxy-functionalized derivatives of specific terpenoids are sometimes desired for their specific bioactivities. Naturally, such derivatives only occur in low amounts and both their isolation and their chemical synthesis are rather uneconomical. Thus, *in vivo* enzymatic transformation of toxic monoterpenoids by heterologously expressed cytochrome P450 monooxygenases as an alternative is under study. As an example, the conversion of limonene to perillic acid (Mirata et al. 2009; van Beilen et al. 2005) and the hydroxylation of 1,8-cineole (Mi et al. 2016) was achieved. In addition, another class of terpenoids, the carotenoids zeaxanthin and *β*-carotene, has been successfully produced in *P. putida* (Beuttler et al. 2011; Loeschcke et al. 2013). In this context, the astonishing metabolic plasticity of *P. putida* was demonstrated by reshaping the native, cyclical EDEMP pathway into a linear EMP pathway by heterologous expression of glycolytic genes from *E. coli*. This led to increased levels of PYR and G3P, both precursors of carotenoid production, resulting in 1.3-fold increase in carotenoid production compared with the parental strain (Sánchez-Pascuala et al. 2019). Further increase of precursor supply seems to be a crucial factor for efficient production.

### Polyketides and non-ribosomal peptides

**Polyketides (**PKs) and non-ribosomal peptides (NRPs) display diverse groups of natural products that have commonly medically relevant activities. Both are assembled by condensation of simple carboxylic or amino acid building blocks. The produced polymers can be cyclized and decorated to form the final product. The PKs 2,4-diacetylphloroglucinol (Martinez et al. 2004) and the UV-protective pigment flaviolin (Gross et al. 2006a) were successfully produced in *P. putida*. An interesting PK/NRP hybrid compound is the antibiotic prodigiosin, due to its anticancer and immunosuppressant activities (Domröse et al. 2015). In *E. coli* prodigiosin transcription is inhibited (Danevčič et al. 2016). Yet, this is not the case for *P. putida*. The TREX expression system, which includes elements of transposon Tn*5*, enabled the random chromosomal integration of the *pig* gene cluster, derived from the native producer *Serratia marcescens* W83. Remarkably, a higher titer could be reached using a strong intrinsic chromosomal promoter (Domröse et al. 2015) compared with utilization of a synthetic T7 RNA polymerase-dependent promoter (Loeschcke et al. 2013). This study led to the discovery of rRNA promoters as strong native promoters for heterologous expression of biosynthetic gene clusters in *P. putida* (Domröse et al. 2019). Furthermore, through the expression of myxobacterial hybrid systems, the antibiotics myxochromide S (Stephan et al. 2006) and tubulysin (Chai et al. 2012) could be synthesized. Since many of the biosynthetic gene clusters required for the production of natural products are larger than 6 kb (Loeschcke and Thies 2015), sophisticated tools are needed for chromosomal integration. One such tool is the recent developed RecET-based markerless recombineering system for *P. putida* (Choi and Lee 2020). The site-specific integration of a 7.4-kb violacein cluster could be shown (Choi et al. 2018). Moreover, the first synthetic polyketide synthase-like pathway for the production of the omega-3 fatty acid docosahexaenoic acid (DHA) was successfully expressed in *P. putida* KT2440 (Gemperlein et al. 2016).

### Recombinant protein production

The expression of antibodies in *P. putida* KT2440 could be achieved with proper folding and promising yields (Dammeyer et al. 2011; Jiménez et al. 2015). In addition, whole-cell *P. putida* or isolated enzymes thereof find actual industrial application (Poblete-Castro et al. 2012a; Rehm 2009; Tiso et al. 2014). An example company is DSM (The Netherlands), which has reported to produce chiral compounds using isolated enzymes from P. putida ATCC 12633 (Hermes et al. 1993; Rehm 2009). Whole-cell *P. putida* biocatalysts are enabled to produce 5-cyanopentanamide (DiCosimo et al. 1996), D-*p*-hydroxyphenyl glycine (Schulze and Wubbolts 1999), and 5-methylpyrazine-2-carboxylic acid (Kiener 1992). Furthermore, strain BIRD-1 is commercially available as plant growth-promoting bacterium (Fosfogel, Bio-Iliberis R&D, Granada, Spain).

## Conclusion and outlook

The choice of microbial hosts for biotechnological applications had long been based on historical tradition (Calero and Nikel 2019) rather than on the bacterial *chassis* (platform) which meets the desired process criteria in the best possible way. This is mainly because microorganisms that have been extensively investigated and characterized can be more easily manipulated to maximize production, and concurrently their behavior during industrial scale-up is more predictable. Recent advantages in synthetic and systems biology tools support turning the tide of the choice of microbial host for industry (Wehrs et al. 2019). This is especially true for *P. putida*. Long known for its versatile metabolism and low nutritional requirements (Timmis 2002), recent advances in omics technologies now enable the discovery of new attractive features of representatives by genome sequencing, the decryption of the central core and energy metabolism by flux analysis (Kohlstedt and Wittmann 2019; Nikel et al. 2015), and the regulation of gene expression on the transcriptional and translational levels. Findings thereof, together with improvements of genetic engineering tools, are of particular value to complete our holistic understanding of *P. putida* and ease the development of superior industrial strains. Moreover, it should be stressed that the capability of producing value-added chemicals from alternative feedstocks (Kohlstedt et al. 2018; Poblete-Castro et al. 2014; Salvachúa et al. 2020a; Wierckx et al. 2012) is a superior feature to pass into the envisaged circular bioeconomy. Beyond the wide product range already demonstrated for *P. putida*, a promising upcoming approach is re-designing the biochemical portfolio by introducing complete synthetic pathways (bio-bricks) to access new-to-nature products, such as halogenated molecules and boron-containing structures in the near future (Nieto-Domínguez and Nikel 2020; Nikel and de Lorenzo 2018). This will open a full new branch of biotechnologically producible molecules and represents an important step regarding the limited fossil-based resources. However, as in the case of other microbes (Wehrs et al. 2019), despite extensive studies and demonstration of feasibility of production on a small scale, large-scale industrial applications of *P. putida* do not yet add up in numbers, with PHA, FDCA, and rhamnolipid production as rare exceptions, as discussed previously. Nevertheless, successful proof-of-concept studies and product life cycle assessments are important starting points for subsequent process optimization and development of viable business cases. But more importantly, they underline the extraordinary metabolic versatility and robustness of *P. putida* as serious player, who will ultimately contribute to a greener and more sustainable cradle-to-cradle economy.

## Electronic supplementary material

ESM 1 (PDF 483 kb)
