# Supplementary material for: Industrial biotechnology of Pseudomonas putida: advances and prospects
Source: Appl Microbiol Biotechnol. 2020 Aug 13;104(18):7745–66. doi: 10.1007/s00253-020-10811-9 (PMC7447670; doi:10.1007/s00253-020-10811-9)
Supplement: Supplementary file 1 — (PDF 483 kb) [file 253_2020_10811_MOESM1_ESM.pdf]

Applied Microbiology and Biotechnology

Industrial biotechnology of *Pseudomonas putida*: Advances and prospects

Anna Weimer[1], Michael Kohlstedt[1], Daniel C. Volke[2], Pablo I. Nikel[2], Christoph Wittmann[1]

[1] Institute of Systems Biotechnology, Saarland University, Saarbrücken, Germany

[2] The Novo Nordisk Foundation Center for Biosustainability, Technical University of Denmark, Denmark

*Campus A1.5, 66123 Saarbrücken, Germany, christoph.wittmann@uni-saarland.de, Phone/FAX: +49-681-302-71970/71972.

Supplementary

Table S1: Available draft genomes of *Pseudomonas putida* (Data provided by Pseudomonas Genome DB (pseudomonas.com)). Respective strain, and its assembly level and assembly accession, version status and release year. Strains marked in red will be available within the next release.

Strain	Assembly level	Assembly accession	Version status	Release year
Pseudomonas putida N1R	Chromosome	GCF_900156185.1	latest	2017
Pseudomonas putida LF54	Scaffold	GCF_000390005.2	latest	2013
Pseudomonas putida PD1	Scaffold	GCF_000799625.1	latest	2014
Pseudomonas putida PA14H7	Scaffold	GCF_000800615.1	latest	2014
Pseudomonas putida ATH-43	Scaffold	GCF_001005285.1	latest	2015
Pseudomonas putida H	Scaffold	GCF_001077495.1	latest	2015
Pseudomonas putida JLR11	Scaffold	GCF_001183585.1	latest	2015
Pseudomonas putida ABAC8	Scaffold	GCF_001467305.1	latest	2015
Pseudomonas putida Simmons01	Scaffold	GCF_001542955.1	latest	2016
Pseudomonas putida KB9	Scaffold	GCF_001645635.1	latest	2016
Pseudomonas putida INSali382	Scaffold	GCF_001653615.1	latest	2016
Pseudomonas putida NFIX47	Scaffold	GCF_900104105.1	latest	2016
Pseudomonas putida A514	Scaffold	GCF_001904555.1	suppressed	2016
Pseudomonas putida DZ-C18	Scaffold	GCF_002094795.1	latest	2017
Pseudomonas putida DZ-C20	Scaffold	GCF_002094785.1	latest	2017
Pseudomonas putida DZ-F23	Scaffold	GCF_002094775.1	latest	2017
Pseudomonas putida DPA1	Scaffold	GCF_002891885.1	latest	2018
Pseudomonas putida PICP2	Scaffold	GCF_003263425.1	latest	2018
Pseudomonas putida HAMBI_6	Scaffold	GCF_003350225.1	latest	2018
Pseudomonas putida LD6	Scaffold	GCF_003586135.1	latest	2018
Pseudomonas putida 12917	Scaffold	GCF_003721355.1	latest	2018
Pseudomonas putida 142223	Scaffold	GCF_003721325.1	latest	2018
Pseudomonas putida JUb85	Scaffold	GCF_004345845.1	latest	2019
Pseudomonas putida II-2	Scaffold	GCF_004357765.1	latest	2019
Pseudomonas putida FF4	Scaffold	GCF_007049805.1	latest	2019
Pseudomonas putida B2017	Scaffold	GCF_007279645.1	latest	2019
Pseudomonas putida n4t	Scaffold	GCF_009078365.1	latest	2019
Pseudomonas putida BOE100	Scaffold	GCF_009746605.1	latest	2019
Pseudomonas putida B001	Contig	GCF_000285395.1	latest	2011
Pseudomonas putida Idaho	Contig	GCF_000226475.1	latest	2011
Pseudomonas putida S11	Contig	GCF_000292775.1	suppressed	2012
Pseudomonas putida CSV86	Contig	GCF_000319305.1	latest	2012
Pseudomonas putida LS46	Contig	GCF_000294445.2	latest	2013
Pseudomonas putida TRO1	Contig	GCF_000367825.1	latest	2013
Pseudomonas putida MTCC 5279	Contig	GCF_000411615.1	latest	2013
Pseudomonas putida S610	Contig	GCF_000497385.1	latest	2013
Pseudomonas putida OUS82	Contig	GCF_000507325.1	latest	2013
Pseudomonas putida T2-2	Contig	GCF_000710785.1	latest	2014
Pseudomonas putida MC4-5222	Contig	GCF_000729805.1	latest	2014
Pseudomonas putida YKD221	Contig	GCF_000787655.1	latest	2014
Pseudomonas putida SQ1	Contig	GCF_000802565.1	latest	2014
Pseudomonas putida KF703	Contig	GCF_000875995.1	latest	2015

Pseudomonas putida UASWS0946	Contig	GCF_000878325.1	latest	2015
Pseudomonas putida SJ3	Contig	GCF_000478865.2	latest	2015
Pseudomonas putida KG-4	Contig	GCF_000987155.1	latest	2015
Pseudomonas putida CBB5	Contig	GCF_001006135.1	latest	2015
Pseudomonas putida SF1	Contig	GCF_001027965.1	latest	2015
Pseudomonas putida 791_PPUT	Contig	GCF_001066335.1	latest	2015
Pseudomonas putida PCL1760	Contig	GCF_001282125.1	latest	2015
Pseudomonas putida IOFA1	Contig	GCF_001293025.1	latest	2015
Pseudomonas putida JCM 18452	Contig	GCF_001312165.1	latest	2015
Pseudomonas putida JCM 18798	Contig	GCF_001312185.1	suppressed	2015
Pseudomonas putida JCM 9802	Contig	GCF_001312145.1	suppressed	2015
Pseudomonas putida HB13667	Contig	GCF_001306495.1	latest	2015
Pseudomonas putida HB4184	Contig	GCF_001306515.1	latest	2015
Pseudomonas putida JQ581	Contig	GCF_001630725.1	latest	2016
Pseudomonas putida B6-2	Contig	GCF_000226035.2	latest	2016
Pseudomonas putida CBF10-2	Contig	GCF_001644955.1	latest	2016
Pseudomonas putida SJTE-1	Contig	GCF_000271965.2	latest	2016
Pseudomonas putida PSP1	Contig	GCF_001700665.1	latest	2016
Pseudomonas putida PSP2	Contig	GCF_001700695.1	latest	2016
Pseudomonas putida PSP3	Contig	GCF_001700655.1	latest	2016
Pseudomonas putida PSP4	Contig	GCF_001700705.1	latest	2016
Pseudomonas putida IAC-RBcr5	Contig	GCF_001750465.1	latest	2016
Pseudomonas putida isolate *Pseudomonas putida* FW305-E2	Contig	GCF_900095365.1	latest	2016
Pseudomonas putida GM4FR	Contig	GCF_001941965.1	latest	2017
Pseudomonas putida ASAD	Contig	GCF_001976065.1	latest	2017
Pseudomonas putida 1312	Contig	GCF_002157515.1	latest	2017
Pseudomonas putida UV4	Contig	GCF_002165695.1	latest	2017
Pseudomonas putida UV4/95	Contig	GCF_002165665.1	latest	2017
Pseudomonas putida FDAARGOS_409	Contig	GCF_002554535.1	latest	2017
Pseudomonas putida FDAARGOS_376	Contig	GCF_002588485.1	latest	2017
Pseudomonas putida CA-3	Contig	GCF_002810225.1	latest	2017
Pseudomonas putida KH-18-2	Contig	GCF_002906815.1	latest	2018
Pseudomonas putida KH-20-11	Contig	GCF_002906795.1	latest	2018
Pseudomonas putida KH-21-114	Contig	GCF_002906835.1	latest	2018
Pseudomonas putida KH-21-134	Contig	GCF_002906855.1	latest	2018
Pseudomonas putida KT-27	Contig	GCF_002906715.1	latest	2018
Pseudomonas putida KT-90	Contig	GCF_002906755.1	latest	2018
Pseudomonas putida KCJK7911	Contig	GCF_003053335.1	latest	2018
Pseudomonas putida KCJK7916	Contig	GCF_003053385.1	latest	2018
Pseudomonas putida NCTC10936	Contig	GCF_900455645.1	latest	2018
Pseudomonas putida NCTC7914	Contig	GCF_900455695.1	latest	2018
Pseudomonas putida NCTC912	Contig	GCF_900455605.1	latest	2018
Pseudomonas putida ATCC 31483	Contig	GCF_003417615.1	latest	2018
Pseudomonas putida ZKA37	Contig	GCF_003550095.1	latest	2018
Pseudomonas putida BIGb0473	Contig	GCF_003752325.1	latest	2018

Pseudomonas putida FDAARGOS_516	Contig	GCF_003938945.1	latest	2018
Pseudomonas putida JR16	Contig	GCF_004519745.1	latest	2019
Pseudomonas putida KB3	Contig	GCF_004614175.1	latest	2019
Pseudomonas putida OR45a	Contig	GCF_004614155.1	latest	2019
Pseudomonas putida NCTC13185	Contig	GCF_901482375.1	latest	2019
Pseudomonas putida NRRL B-251	Contig	GCF_005930605.1	latest	2019
Pseudomonas putida NRRL B-252	Contig	GCF_005930545.1	latest	2019
Pseudomonas putida TUM15504	Contig	GCF_009012945.1	latest	2019
Pseudomonas putida ODNR4SY	Contig	GCF_009905395.1	latest	2020

Table S2: Available full genomes of *Pseudomonas putida* (Data provided by Pseudomonas Genome DB (pseudomonas.com), Isolation source from NCBI BioSample Database, Accessed 05/20/2020). Respective strain, and its assembly level and assembly accession, version status, isolation source and release year. Strains marked in red will be available with the next release. For creating Figure 3, the year of the first assembly version was considered, versions marked in blue were not taken into account.

**Strain**	**Assembly level**	**Assembly accession**	**Version status**	**Release year**	**isolation_source**
Pseudomonas putida W5	Complete Genome	GCF_009883635.1	latest	2020	alfalfa plant root nodules
Pseudomonas putida W619	Complete Genome	GCF_000019445.1	latest	2008	black cottonwood tree
Pseudomonas putida HB3267	Complete Genome	GCF_000325725.1	latest	2012	clinical (human)
Pseudomonas putida H8234	Complete Genome	GCF_000410575.1	latest	2013	clinical (human)
Pseudomonas putida GB-1	Complete Genome	GCF_000019125.1	latest	2008	fresh water
Pseudomonas putida ND6	Complete Genome	GCF_000264665.2	latest	2012	industrial wastewater
Pseudomonas putida JYR-1	Complete Genome	GCF_008605605.1	latest	2019	oil contaminated soil
Pseudomonas putida JB	Complete Genome	GCF_001767335.1	latest	2016	PCB contaminated soil
Pseudomonas putida 1290	Complete Genome	GCF_005080685.1	latest	2019	pear phyllosphere
Pseudomonas putida F1	Complete Genome	GCF_000016865.1	latest	2007	polluted creek
Pseudomonas putida S13.1.2	Complete Genome	GCF_000498395.2	latest	2015	rhizosphere soil
Pseudomonas putida AA7	Complete Genome	GCF_002025705.1	latest	2017	root
Pseudomonas putida PC2	Complete Genome	GCF_001636055.1	latest	2016	seeds
Pseudomonas putida KT2440	Complete Genome	GCF_000007565.1	replaced	2002	soil
Pseudomonas putida BIRD-1	Complete Genome	GCF_000183645.1	latest	2010	soil
Pseudomonas putida DLL-E4	Complete Genome	GCF_000691565.1	latest	2014	soil
Pseudomonas putida S12	Complete Genome	GCF_000495455.2	latest	2014	soil
Pseudomonas putida 1A00316	Complete Genome	GCF_001515585.1	latest	2016	soil
Pseudomonas putida KT2440 (TIGR)	Complete Genome	GCF_000007565.2	latest	2016	soil
Pseudomonas putida KF715	Complete Genome	GCF_002356095.1	latest	2016	soil

Pseudomonas putida JBC17	Complete Genome	GCF_003228315.1	latest	2018	soil
Pseudomonas putida NX-1	Complete Genome	GCF_003290365.1	latest	2018	soil
Pseudomonas putida B4	Complete Genome	GCF_003671955.1	latest	2018	soil
Pseudomonas putida B1	Complete Genome	GCF_007833495.1	latest	2019	soil
Pseudomonas putida S16	Complete Genome	GCF_000219705.1	latest	2011	soil from field under continuous tobacco cropping
Pseudomonas putida E41	Complete Genome	GCF_002736125.1	latest	2017	surface sterilized roots of sida hermaphrodita
Pseudomonas putida E46	Complete Genome	GCF_002736045.1	latest	2017	Surface sterilized roots of sida hermaphrodita
Pseudomonas putida NBRC 14164	Complete Genome	GCF_000412675.1	latest	2013	unknown
Pseudomonas putida NCTC13186	Complete Genome	GCF_900636645.1	latest	2018	unknown
Pseudomonas putida PP112420	Complete Genome	GCF_001886975.1	latest	2016	Urine (human)
Pseudomonas putida DOT-T1E	Complete Genome	GCF_000281215.1	latest	2012	water from wastewater treatment plant
Pseudomonas putida IEC33019	Complete Genome	GCF_002741075.1	latest	2016	unknown
